# Fabrication of µFFE Devices in COC via Hot Embossing with a 3D-Printed Master Mold

**DOI:** 10.3390/mi14091728

**Published:** 2023-09-02

**Authors:** Matthew B. LeMon, Cecilia C. Douma, Gretchen S. Burke, Michael T. Bowser

**Affiliations:** Department of Chemistry, University of Minnesota, Minneapolis, MN 55455, USA

**Keywords:** micro free-flow electrophoresis, microfluidics, cyclic olefin copolymer, hot embossing, 3D printing

## Abstract

The fabrication of high-performance microscale devices in substrates with optimal material properties while keeping costs low and maintaining the flexibility to rapidly prototype new designs remains an ongoing challenge in the microfluidics field. To this end, we have fabricated a micro free-flow electrophoresis (µFFE) device in cyclic olefin copolymer (COC) via hot embossing using a PolyJet 3D-printed master mold. A room-temperature cyclohexane vapor bath was used to clarify the device and facilitate solvent-assisted thermal bonding to fully enclose the channels. Device profiling showed 55 µm deep channels with no detectable feature degradation due to solvent exposure. Baseline separation of fluorescein, rhodamine 110, and rhodamine 123, was achieved at 150 V. Limits of detection for these fluorophores were 2 nM, 1 nM, and 10 nM, respectively, and were comparable to previously reported values for glass and 3D-printed devices. Using PolyJet 3D printing in conjunction with hot embossing, the full design cycle, from initial design to production of fully functional COC µFFE devices, could be completed in as little as 6 days without the need for specialized clean room facilities. Replicate COC µFFE devices could be produced from an existing embossing mold in as little as two hours.

## 1. Introduction

Micro free-flow electrophoresis (µFFE) is a microscale, continuous flow, separation technique first demonstrated by Raymond et al. in 1994 [1]. In µFFE, pressure is used to move fluid through a narrow planar channel under laminar flow conditions. An electric field is applied perpendicularly to the pressure-driven flow causing lateral separation of analytes based on their electrophoretic mobility. Since its inception, µFFE has been used for a wide variety of applications, such as: micro-scale sample purification [2,3,4,5,6,7], high-efficiency 2D separations (e.g., LC × µFFE [8,9,10] and CE × µFFE [11]), on-line reaction monitoring [5,12,13], binding assays [14], and aptamer selection [15]. Furthermore, µFFE has been used to assess a wide range of analytes, including amino acids [11], fluorescent dyes [16,17,18,19], proteins [8,11,19,20], bacteria [7], and organelles [21].

µFFE devices have been fabricated using a wide variety of methods, including wet etching [16,22], computer numeric control (CNC)-milling [23,24], injection molding [25], multistep liquid-phase lithography [26], and multi-step lamination [27,28]; in a broad range of materials including silicon [1], glass [16], poly(dimethyl siloxane) (PDMS) [29], poly(methyl methacrylate) (PMMA) [30], acrylonitrile butadiene styrene (ABS) [19], and cyclic olefin polymer (COP) [25]. Each material presents its own advantages and disadvantages in terms of sensitivity, separation efficiency, chemical compatibility, and ease of fabrication. Glass, for example, is an optically transparent, rigid material that is stable over a wide range of pH, while being relatively chemically inert. However, glass devices are relatively expensive and slow to produce, requiring a specialized fabrication facility for much of the process. Furthermore, glass µFFE devices often require frequent treatment to ensure consistent surface chemistry and prevent surface adsorption [9].

In contrast, the fabrication of microfluidic devices in PDMS is fast and inexpensive [29,31,32]. However, the elasticity of the material combined with the low aspect ratio of the separation channel in µFFE devices requires special design considerations such as posts that can decrease separation space and efficiency [31]. The hydrophobicity of PDMS creates additional challenges, making bubble-free filling by aqueous solvents difficult, and allowing hydrophobic analytes to readily adsorb to the surface. The hydrophobic surface also complicates the removal of bubbles that form at the electrodes of a µFFE device due to electrolysis, which can quickly accumulate and disrupt flow [33]. To avoid these issues, the hydrophilicity of the surface can be modified using a variety of methods; however, PDMS can quickly recover its hydrophobicity due in part to its low glass transition temperature [34,35].

Rigid polymers like PMMA, ABS, COP, and cyclic olefin copolymer (COC) provide a compromise between glass and PDMS, being less expensive and faster to produce than glass devices with better performance characteristics than PDMS [19,23,25,27]. COC has seen increased usage as a material to produce microfluidic devices over the past decades due to its many desirable properties. In addition to being inexpensive and rigid, it has both high optical clarity and low background fluorescence, allowing highly efficient sample detection [36]. COC is also resistant to acids, bases, and most polar organic solvents such as acetone and methanol. COC boasts extremely low water absorption (<0.01%), an order of magnitude less than PMMA, which results in humidity having little to no effect on reproducibility [37]. However, like many plastics, the surface of COC is hydrophobic, and like PDMS can require surface modification to be suitable for protein analysis [38].

COC has several variations in microstructure, each with different thermal resistances and viscosities that can be selected based on the desired application [39]. Combined with its low density, this makes COC an excellent material for fabrication methods like injection molding and hot embossing, where its low viscosity improves fidelity. As a result, hot embossing and injection molding are the primary methods used for fabricating COC microfluidic devices [36,37,40]. While injection molding is capable of higher throughput than hot embossing due to its shorter cycle times, higher mold costs and production time make it poorly suited for prototyping. In addition, hot embossing provides better replication accuracy for microstructures, less shrinkage, and less warping compared to injection molding [41]. To our knowledge hot embossing has yet to be explored as an option for µFFE device fabrication.

Hot embossing is a process in which a polymer is heated above its glass transition temperature, and a mold is then pressed into it, imprinting features into the polymer. Master molds for this process can be produced in a wide range of materials using a variety of lithography or direct structuring methods [42,43]. One particular lithography method frequently used for structuring master molds in silicon is deep reactive ion etching (DRIE) [37]. This technique is exceptional at producing high aspect ratio structures, but defects in the wafer and uneven etch rates make DRIE non-ideal for larger scale µFFE designs that cover several square centimeters [44]. Combined with the cost of operating and maintaining DRIE equipment and the need for clean room facilities, this method of mold fabrication is poorly suited for µFFE device development where quick iteration of designs is desired.

PolyJet 3D printing provides an excellent inexpensive alternative to DRIE for the fabrication of larger mold designs, as it does not require specialized instrumentation and clean room facilities. PolyJet 3D printing relies on jetting microdroplets of photocurable polymer from ink-jet style print heads followed by rapid curing with UV lamps attached to the 3D print cartridge [45]. PolyJet 3D printing can produce layer heights as low as 14–28 µm. Combination with computer-aided design (CAD) software allows rapid iteration of new designs. 3D printing also provides easy control over the taper of the mold, which is particularly important for demolding when hot embossing [46]. With regards to µFFE, PolyJet 3D printing provides a viable means of rapidly producing and iterating mold designs for hot embossing.

In this manuscript, we assess the potential of hot embossing to produce high-performance, low-cost, µFFE devices in COC. Optimization of fabrication steps including device design, embossing mold preparation, embossing/de-embossing temperatures, and solvent-assisted bonding is discussed. Separation performance and limits of detection of COC µFFE devices are assessed using the separation of a standard mixture of fluorescent dyes. While other methods for inexpensive, rapid µFFE production exist, they are not well suited for fabrication in COC or do not have the same potential for high-fidelity, high-throughput fabrication [19,29,30]. The use of a PolyJet 3D printer will also be assessed as a means of producing hot embossing master molds to allow faster prototyping of COC devices without the need for expensive nanofabrication techniques or design testing in alternative materials.

## 2. Experimental

### 2.1. Materials

Solutions were made with deionized water (18.3M, Milli-Q; Millipore, Bedford, MA, USA) and filtered through a 0.22 μm nitrocellulose membrane (Millipore). μFFE separation buffer was 25 mM HEPES (Sigma-Aldrich, St. Louis, MO, USA) and 300 μM Triton X-100 (Sigma-Aldrich) with the pH adjusted to 7.00. Fluorescent dye solutions consisted of fluorescein, rhodamine 110 chloride, and rhodamine 123 (Sigma-Aldrich) dissolved in a separation buffer. Verowhite resin (Sculpteo Inc., Oakland, CA, USA) was used to 3D print the master mold. Silastic RTV-3110 (Sigma-Aldrich) catalyzed by Xiameter RTV-3010S (Sigma-Aldrich) was used to cast a negative of the master mold. Weicon C (Weicon, Kitchener, ON, Canada) was used to make the embossing molds. Transparent acrylonitrile-butadiene-styrene (ABS) (Matterhackers, Lake Forest, CA, USA) was used to 3D print the bonding rigs. TOPAS 6017s-04 cyclic olefin copolymer (COC, 6” × 6” × 0.040” molded plates) was used to make the devices (TOPAS, Raunheim, Germany), and was cut to the desired size using a Universal Systems PLS6.150D Laser Cutter (Universal Laser Systems, Scottsdale, AZ, USA). The electrodes for the COC devices were 100 µm diameter platinum wire (Sigma-Aldrich). Quickset Epoxy (Loctite, Westlake, OH, USA) was used to secure ports and seal the electrodes into the device. Cyclohexane (Fisher Scientific, Hampton, NH, USA) was used to make cyclohexane vapor for clarifying and bonding the COC halves. Crystal Bond (Electron Microscopy Sciences, Hatfield, PA, USA) was used to seal the capillary in the COC devices.

### 2.2. COC μFFE Device Fabrication

**Figure 1:**
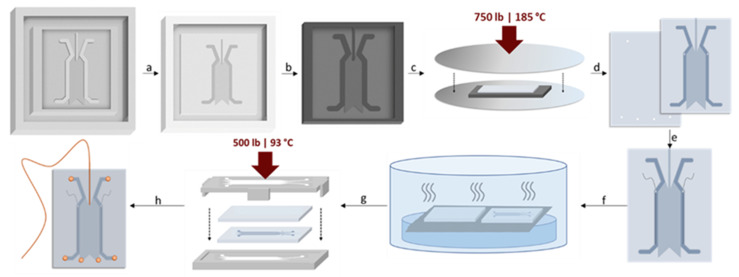
Schematic of µFFE device fabrication in COC using hot embossing. (**a**) Cast master mold in silicone. (**b**) Recast silicone mold in Weicon C. (**c**) Hot embossing. (**d**) Drill inlet, outlet, and electrode holes. (**e**) Attach electrodes. (**f**) Cyclohexane vapor exposure. (**g**) Bond under heat and pressure in the bonding rig. (**h**) Attach buffer ports and sample capillary.

Figure 1 shows an overview of the µFFE hot embossing fabrication process. The master mold was designed in Autodesk Inventor and printed on a Stratasys J750 PolyJet 3D printer using default high mix mode settings for general-purpose VeroPureWhite at 27 µm resolution (University of Minnesota Medical Device Center, Minneapolis, MN, USA). The CAD file was a negative of the final imprinted design and included a 28 μm tall × 2.5 cm long × 1 cm wide separation channel, as well as 200 μm tall electrode channels, and a 400 μm tall × 400 μm wide capillary inlet channel. The separation channel was smoothed using wet sand with 1500 grit followed by 3000 grit sandpaper to remove print lines and reduce the channel height. The mold was cast in Xiameter RTV-3010S catalyzed Silastic RTV-3110 and allowed to cure overnight before careful separation. The Silastic mold was then cast in Weicon C polymer (Weicon, Kitchener, ON, Canada) and was backed with an aluminum block wrapped in heavy-duty aluminum foil. After curing at room temperature for 48 h, the block and foil were removed, and any excess polymer was chipped away. The mold was then tempered to withstand embossing conditions by heating on a hotplate at 40–120 °C over 18 h, increasing the temperature by 20 °C each hour until reaching 120 °C.

Hot embossing was performed on a Carver thermal press with an embossing temperature of 185 °C, a de-embossing temperature of 173 °C, and a load of 750 lb. Prior to embossing, the COC polymer was cleaned with acetone to remove any residual compounds from the surface and dried using compressed air. The Weicon C mold was aligned with a 5.8 cm × 4.5 cm × 1 mm thick piece of COC between two flat, mirror-polished, steel plates before being placed in the pre-heated thermal press. The mold and polymer were then allowed to reach thermal equilibrium at the embossing temperature (173 °C) over approximately 60 s before the load was slowly applied over 15 s. The press was then cooled to the de-embossing temperature where the load was released, and the mold and imprinted polymer were separated.

Imprints were placed feature side down and propped approximately 1 mm off a wire mesh stage above a 150 mm Petri dish filled with 100 mL of cyclohexane. This was then sealed using an inverted crystalizing dish, exposing the imprints to cyclohexane vapors at room temperature for 28 min to smooth and clarify the imprinted COC wafers. The imprints were then allowed to dry completely.

Wire electrodes were inserted into 0.4 mm holes drilled at a 30° angle at the top of each electrode channel. These wires were then embedded in the electrode channels by melting them into the COC with a soldering iron. A second 5.8 cm × 4.5 cm × 1 mm thick piece of COC had two 1 mm buffer inlet and four 1 mm buffer outlet holes drilled into it.

The surfaces of the two COC pieces were activated for bonding using cyclohexane vapor in the exposure chamber described above. The non-imprinted and imprinted halves were exposed face-down at room temperature for 40 and 12 min, respectively. The halves were then aligned in a 3D-printed ABS bonding rig, placed into a Carver thermal press at 93 °C, and put under a 500 lb load for 20 min, at which point the load was released and the bonded device was removed from the rig.

**Figure 2:**
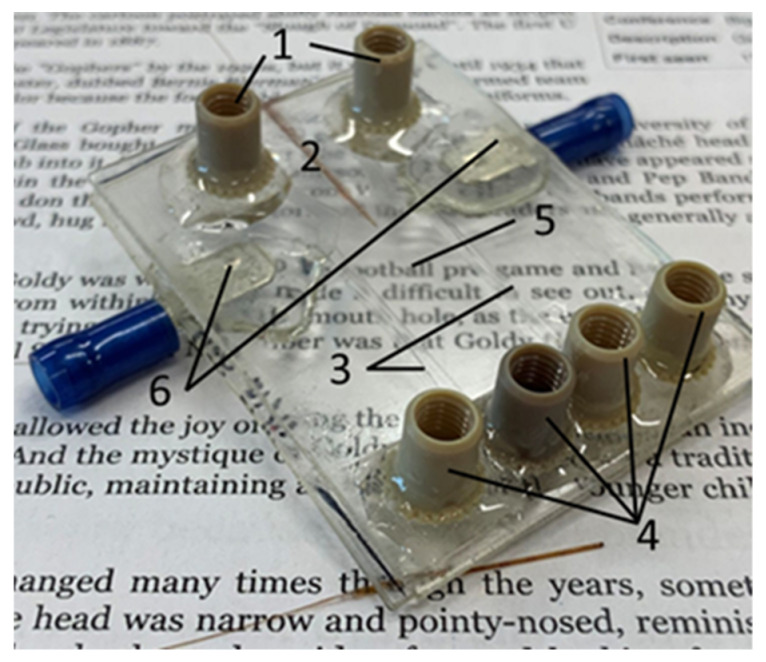
Image of a fully fabricated COC µFFE device. Labels show the (1) buffer inlets, (2) sample inlet, (3) electrode channels (2 mm wide, 200 µm deep), (4) buffer outlets, (5) separation channel (1 cm wide, 2.5 cm long, 55 µm deep), and (6) electrical connections.

The electrode holes of the device were sealed using quickset epoxy. The PEEK nano-ports (Upchurch Scientific, Oak Harbor, WA, USA) were applied to the buffer inlet and outlet ports using quickset epoxy. A 150 µm o.d., 20 µm i.d. fused silica capillary (Polymicro Technologies, Phoenix, AZ, USA) was positioned in the capillary channel. The crystal bond was then melted at 125 °C on a hot plate and drawn into the capillary channel by vacuum. Electrical connections were fixed to the device using quickset epoxy, soldered to the exposed wires, and the wires were coated in epoxy to avoid accidental damage. A fully fabricated COC µFFE device is shown in Figure 2.

### 2.3. μFFE Separations

Tefzel Tubing (1/16 in. o.d. × 0.040 in. i.d., IDEX Health and Science, Oak Harbor, WA, USA) and PEEK fittings and ferrules (IDEX Health and Science, Oak Harbor, WA, USA) were used to interface with the buffer inlets and outlets. Separation buffer (25 mM HEPES, 300 μM Triton X-100, pH = 7.00) was pumped into the device through the buffer inlets using a syringe pump at 0.5 mL/min per channel (Model #55-2222, Harvard Apparatus, Holliston, MA, USA). A three-dye mixture was pumped into the device through the sample inlet capillary using a syringe pump at 0.5 μL/min (Model #70-2213 Pico Plus, Harvard Apparatus), interfacing with the capillary via a zero dead volume connector. During separations, a potential was applied to the left electrode using a power supply (Model PS310/1250V-25W, Stanford Research Systems, Sunnyvale, CA, USA) at a set value between 0 and 150 V. The right electrode was connected to the ground.

### 2.4. Laser-Induced Fluorescence Detection and Data Collection

Laser-induced fluorescence (LIF) was performed using a 30-mW beam from a 150-mW diode pumped 488 nm solid-state laser (Sapphire, Coherent, Santa Clara, CA, USA), which was broadened into an ~1.5 cm wide by ~150 μm thick line across the separation channel directly under the detection setup. LIF detection was performed at 1× zoom using an AZ 100 stereomicroscope (Nikon Corporation, Tokyo, Japan) mounted with a Cascade 512B CCD camera (Photometrics, Tucson, AZ, USA). The microscope was fitted with a filter cube that had a 470/40 nm excitation filter, 525/50 nm emission filter, and a 495 nm cutoff dichroic mirror. A 0.5× objective (Nikon Metrology NV, Leuven, Belgium) was used along with a 0.7× CCD lens for detection. Images were acquired at a rate of 5 Hz with a gain of 1600. The detection setup was enclosed in a light-tight enclosure (Newport, Irving, CA, USA) within a darkroom. Line scans of samples and blanks were exported as a text file and processed using a custom MATLAB script.

### 2.5. μFFE Depth Profiling

The feature depths of hot-embossed COC wafers were profiled before and after cyclohexane vapor exposure using a KLA-Tencor P7 surface profiler (KLA, Milpitas, CA, USA) with a scanning speed of 100 μm/s and a total scan distance of 15 mm. The imprints were flattened prior to profiling to ensure the P7 stage vacuum would hold them by heating them to 163 °C in a thermal press between two flat, mirror-polished, steel plates under a load of 500 lbs. The press was cooled to 108 °C and the imprints were removed and allowed to cool to room temperature.

## 3. Results and Discussion

Our goal was to develop a fabrication method to produce high-performance µFFE devices at low cost while maintaining the potential for rapid prototyping and design iteration. Hot embossing was chosen as a fabrication approach that enabled production in COC, a desirable polymeric substrate with high clarity, broad chemical resistance, and low water absorption. Master molds were produced using 3D printing, allowing control over important features such as tapered edges, and enabling rapid turnaround in the design cycle. The overall design of the hot embossed COC µFFE devices was similar to those previously described by our group in glass and 3D-printed device designs with one significant difference. In previous designs [18,19], the electrode channels were 4× deeper than the separation channel resulting in 16-fold higher linear velocity in the electrode channel in comparison to the separation channel. These deeper electrode channels efficiently eliminate electrolysis bubbles from the device, ensuring stable buffer flow [18]. With COC being more hydrophobic than glass, and therefore more likely to retain bubbles, the electrode channel was designed to be 10× deeper resulting in 100-fold faster linear velocity in the electrode channel than the separation channel.

### 3.1. Mold Optimization

Using a PolyJet 3D-printed master mold presented unique benefits and limitations. In addition to being faster and cheaper than traditional mold fabrication methods, the use of 3D printing allows for rapid changes to the master mold design with significant control over the taper of feature edges. Insufficient taper causes the mold and polymer to lock together during de-embossing, causing substantial damage and eventual failure of the Weicon C mold. A limitation of the PolyJet 3D-printed master mold is the variance in the XY axis combined with the staircase pattern of the printed mold in the Z axis. As a result, the 3D prints have rough side walls which require larger tapers to facilitate de-embossing. Our initial designs used a 2° taper that led to mold failure within the first few hot embossing cycles. The taper was increased to 5° which allowed molds to last for roughly 15–30 cycles before naturally wearing down enough to warrant replacement. The use of such high tapers is of little consequence to our structure due to the low aspect ratio of the µFFE separation channel. However, large taper angles present a substantial limitation with regard to high aspect ratio structures, such as the narrow channels often used in microfluidic devices [40,47]. Another major limitation PolyJet 3D printing presents is the artifacts it creates in the form of print lines. These transfer through the process to the device and scatter light, reducing device performance. The separation channel of the master mold was sanded using 1500- and 3000-grit sandpaper until the print lines were no longer visible to minimize the effect of these artifacts. Finally, the inability to emboss directly from the master mold provides a significant limitation, as the need for multiple recasts significantly increases the time to produce a prototype. An alternative mold fabrication technique like micro-milling can overcome this limitation, provided sharp inner corners are not necessary for the device [48].

### 3.2. Embossing Optimization

The embossing and de-embossing temperatures tested were kept within 5–15 °C of COC’s glass transition temperature (178 °C). This range of temperatures was used to reduce thermally induced stress caused by the difference in thermal expansion coefficients between the COC polymer and Weicon C mold [47]. The optimal conditions for the embossing cycle were judged based on several easily observable qualitative factors including COC polymer warping, poor filling around features, Weicon C mold damage, and COC polymer sticking to the Weicon C mold. Poor filling around the mold structures was an indication that the embossing temperature was too low, preventing sufficient COC polymer flow during embossing. Warping of the substrate indicated that the embossing temperature was too high, causing the COC to flow too readily and distort its shape. COC polymer sticking to the mold demonstrated that the de-embossing temperature was too high, as it was still flowing enough to form strings between the mold and imprint when separated. Mold damage was typically a sign that the de-embossing temperature was too low, causing the COC polymer to grip the mold and break off small pieces around the edges of the features. In extreme cases, large pieces would break off, rendering the Weicon C mold unusable. Considering these factors, 185 °C and 173 °C were found to be the optimal temperatures for embossing and de-embossing, respectively. Embossing optimization was performed using a pressure of 750 lbs (i.e., 3.3 kN). This pressure was above the threshold necessary to imprint the mold into the COC substrate at the embossing temperature. The literature review suggests that changes in embossing pressure above this threshold have little impact on the fabrication outcome and no further optimization was performed [41].

### 3.3. Wafer Bonding Optimization

**Figure 3:**
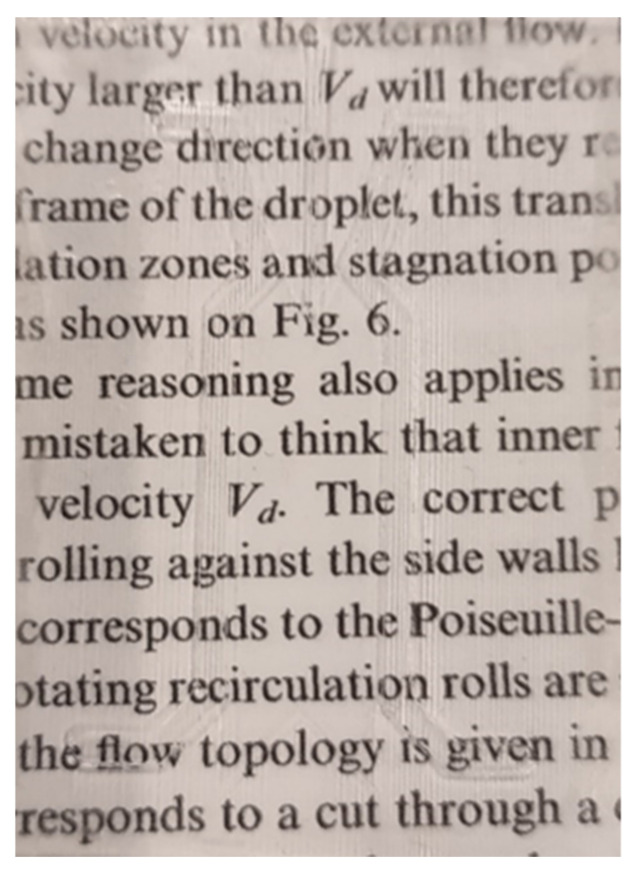
Printed text imaged through an embossed COC wafer demonstrating optical clarity achieved after 28 min of cyclohexane clarification.

After embossing, COC wafers were sealed using solvent vapor-assisted thermal bonding, a commonly used method that uses pressure to hold solvent-activated polymer halves together while heat rapidly drives off the solvent leading to entanglement of polymer chains and the formation of a strong bond [49]. The extent to which each half is activated by the solvent is critically important, as overexposure to the embossed polymer can lead to channel deformation, especially when put under pressure. The low aspect ratio of µFFE designs makes devices particularly prone to overexposure giving rise to sagging and potentially bonding in the separation channel. When optimizing the exposure time for the imprinted wafer, clarification of the COC polymer was used as a qualitative marker (see Figure 3). Clarification was observable after approximately 12 min of cyclohexane vapor exposure at room temperature. As a result, 12 min was used as the exposure time for the imprinted half to ensure activation while minimizing channel deformation during bonding. However, only 12 min of vapor exposure left the separation channel somewhat opaque. To enhance clarity, the imprinted wafer was pre-exposed to cyclohexane vapor for 28 min and allowed to dry completely prior to the 12 min exposure for bonding described above.

Tackiness was used, in addition to bonding efficacy and channel deformation, to optimize the exposure time for the unembossed wafer. Tackiness was characterized by the ability to imprint into and displace the polymer as well as by the two halves sticking together loosely when placed into contact. The polymer halves holding together loosely indicated they were entangling when placed into contact, which occurred after roughly 30 min of cyclohexane vapor exposure. Bonds attempted at this exposure time resulted in incomplete sealing of the device. Overexposure was signified by the ability to imprint into and displace polymer by hand with minimal force, which occurred after approximately 45 min of cyclohexane vapor exposure. Bonds attempted at this exposure time resulted in COC polymer flowing into and distorting the electrode and capillary channels, with the collapse of the separation channel being common. Within this range, 40 min of cyclohexane vapor exposure of the unimprinted wafer was found to produce strong bonds that could not be separated by hand while avoiding collapsing the separation channel. Once sufficiently activated, the imprinted and unimprinted wafers were aligned in a bonding rig and placed in a pre-heated thermal press. Due to the volatility of the solvent, and the consequently short-lived activated state of the COC polymer, properly aligning the wafers in the bonding rig and placing them in the thermal press under bonding conditions within 1 to 2 min was imperative to form a successful bond.

Once the activated wafers were aligned in the thermal press, a load was applied to hold the wafers together while heat was used to drive off the solvent. A 500 lb load was used to hold the COC wafers in firm contact during this step. It was important to keep the temperature high during the bonding step to drive off the solvent quickly and completely, as residual cyclohexane would form pockets between the bonded wafers, weakening the bond and potentially peeling it apart over time. Typically, a solvent bonding temperature would be chosen slightly below the glass transition temperature of the polymer (i.e., 178 °C) [50]. However, our use of a 3D-printed ABS bonding rig to hold the wafers in place during bonding limited the temperature that we could apply to 93 °C. 

The bonding rig featured two halves, each with cutouts of the channel design. One half featured a 5.8 cm × 4.5 cm × 0.5 mm indent to help align the wafers in the rig. The other half featured posts at the center of each side that would hold the two halves of the rig in alignment. The bonding rig was key to the process, directing pressure away from the channels thus reducing the likelihood of bonding in the separation channel, while simultaneously helping to hold the two halves in alignment. The use of a 3D printer for production was significant as it allowed the bonding rig to be quickly and cheaply altered to accommodate changes in the master mold design.

### 3.4. Characterization of Embossing Fidelity 

Once the optimal fabrication conditions had been determined, imprinted COC wafers were evaluated using a KLA Tencor P-7 surface profiler before and after cyclohexane vapor exposure to assess the effect on the channel dimensions and the fidelity of the structures. The effects of sanding the master mold to remove 3D print lines were also determined.

**Figure 4:**
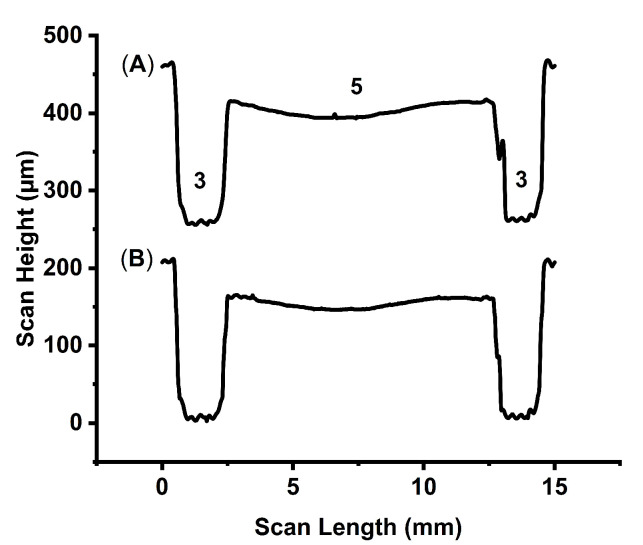
KLA Tencor P7 depth profile recorded across the electrode and µFFE separation channels of an embossed COC wafer (A) before and (B) after 40 min cyclohexane vapor exposure at room temperature. (3) and (5) correspond to the electrode and separation channels, respectively as labelled in Figure 2.

Table 1 and Figure 4 both show that solvent exposure had minimal effects on the channel structures of the device. A comparison of the electrode and separation channels in Figure 4 shows that sanding the master mold minimized 3D printing artifacts in the separation channel of the final embossed COC wafer. The remaining 3D print lines are observable as small, regularly spaced ridges the in the electrode channels. Sanding successfully removed these ridges from the separation channel. In addition to smoothing the separation channel, sanding introduced a 3–4× increase in the variance in channel height relative to the electrode channels. Sanding also creates a distinct valley in the separation channel that affects the flow path of the sample stream, as the stream will follow the deepest portion of the channel. In this geometry, the sample stream flows off-center to the right of the device, as devices are inverted with respect to Figure 4 to avoid inlet and outlet tubing obscuring the separation channel or scattering laser light during detection. While the non-linear flow paths do not have a significant effect on stream width or separation, it does reduce the available separation space for either the negative or positively charged analytes before they forced into the electrode channel depending on electrode polarity. The uneven separation channel could be addressed using profiling to inform further sanding of the master mold to produce a flatter separation channel. Future advances in 3D printer resolution and reduced layer heights may also negate the need for sanding all together.

### 3.5. µFFE Separation and Limits of Detection

**Figure 5:**
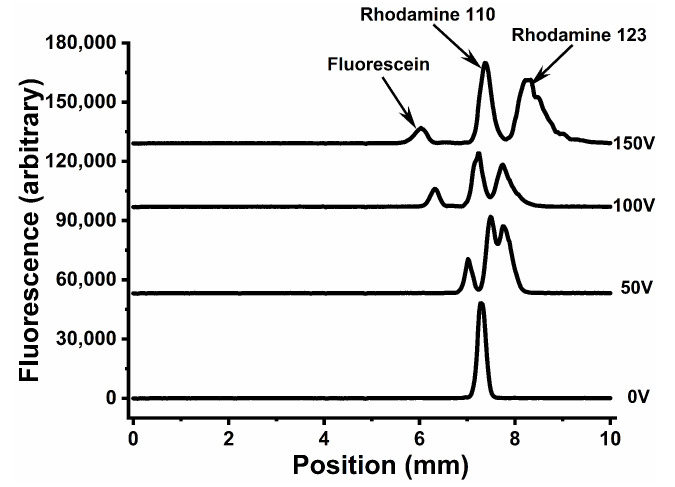
µFFE separation of fluorescein (50 nM), rhodamine 110 (500 nM), and rhodamine 123 (2.5 µM) on a hot embossed COC device using LIF detection. The right electrode was held at ground while 0 to +150V was applied to the left electrode.

**Figure 6:**
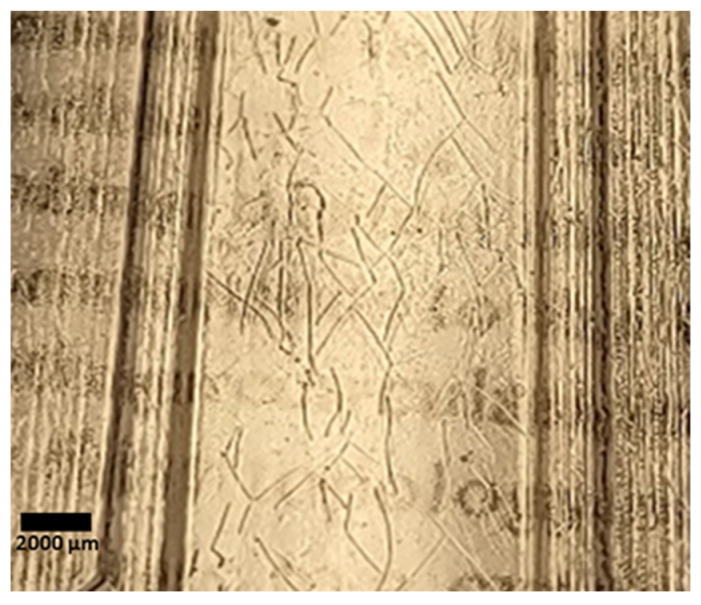
An imprinted COC wafer exposed to cyclohexane vapor for approximately 2 h. Excessive cyclohexane vapor exposure gave rise to visibly observable groove patterns in the COC wafer after solvent evaporation.

Figure 5 shows a µFFE separation of three fluorescent dyes with different charges: fluorescein (negative), rhodamine 110 (neutral), and rhodamine 123 (positive) in the COC device. Baseline resolution was achieved at 150 V. Fabrication defects can be seen giving rise to minor dead spots where fluorescence is lower than expected in the rhodamine 123 peak of the 150 V scan. We believe this is in part an artifact caused by solvent evaporation resulting in semi-crystalline regions that can scatter light. Long cyclohexane exposures accentuate the problem, generating the visible groove patterns shown in Figure 6. While these dead spots do not impact separation, they could potentially be removed with further optimization of the bonding procedure.

Loss of sensitivity due to lower optical transparency and surface smoothness is always a concern when transitioning from glass to polymeric substrates as the fabrication material. To assess the performance of the COC µFFE device, limits of detection (LOD) for the three fluorescent dyes were determined. A stock of the three fluorescent dyes were diluted with separation buffer resulting in five solutions ranging in concentration from 5–500 nM for fluorescein and rhodamine 110, and 50–5000 nM for rhodamine 123. Table 2 shows the LOD recorded on the COC device. Observed LODs were comparable with previously reported devices, showing an approximately 3-fold higher LOD than glass devices, and an approximately 2-fold lower LOD than 3D-printed ABS devices. This improvement in LOD relative to the 3D-printed ABS devices was expected due to the low background fluorescence of COC [36] and the lack of print-lines that are present in the ABS devices.

Longevity was a clear advantage of the COC µFFE devices. COC µFFE devices were regularly operated for periods as long as a year without failing or showing signs of degradation. This is in stark contrast to ABS µFFE devices which, due to cycles of water adsorption and drying, would become opaque or crack within 2–4 weeks of use [19]. COC is known to be resistant to acids, bases, and most polar organic solvents such as acetone and methanol [37]. We routinely rinse our COC µFFE devices with pure methanol with no noticeable effect on channel integrity or clarity. Glass µFFE devices are more robust that those fabricated in ABS, operating for approximately 6–12 months before failure of the gold/titanium electrodes. At this early design stage, we regularly cycle out our COC devices before they fail. We anticipate that they will rival and potentially surpass the longevity of glass due to the thicker electrical connections they possess. In addition, COC µFFE devices only cost approximately $50 per unit in non-recoverable materials to produce. While this is significantly more than the approximate $0.20 it costs to produce an ABS device [19], the COC devices remain substantially cheaper than glass devices and do not require a specialized clean room facility for fabrication. The full design cycle from master mold design to device fabrication could be completed in 6 days, with subsequent fabrication of numerous replicate devices from the embossing mold in as little as two hours. 

## 4. Conclusions

The current manuscript demonstrates the potential of hot embossing for the high throughput production of low-cost µFFE devices in COC with performance metrics competitive with those previously reported in glass or ABS. PolyJet 3D-printed hot embossing master molds offer the ability to rapidly iterate microfluidic designs and produce high-performance devices in desirable materials at low costs without the need for specialized instrumentation or clean room facilities. COC µFFE devices could be produced within six to seven days of acquiring a 3D-printed master mold. With the continual advancement of 3D printing technology improving resolution, decreasing layer heights, and decreasing costs this technique has the potential for widespread use in the rapid iteration and production of new microfluidic designs. Production could be rapidly scaled up thereafter, with each device taking roughly two hours to produce from an existing embossing mold.

## Figures and Tables

**Table 1:** Comparison between device dimensions observed in embossed COC wafers before and after 40 min cyclohexane vapor exposure. Confidence intervals are the standard deviation of the depth measured across the feature in a trace generated from the average of three replicate measurements made on a single imprinted COC wafer.

Feature Depth	Pre-Exposure (μm)	Post-Exposure (μm)
Separation Channel	55 ± 6	56 ± 8
Electrode Channels	203 ± 2	201 ± 2

**Table 2:** Comparison of limits of detection between COC, ABS, and glass µFFE devices.

	Fluorescein	Rhodamine 110	Rhodamine 123
COC Device	2 nM	1 nM	8 nM
Glass Device [19]	0.6 nM	0.3 nM	3 nM
ABS Device [19]	5 nM	2 nM	10 nM

## Data Availability

The data presented in this study are available on request from the corresponding author.
